# The RanGTP Pathway: From Nucleo-Cytoplasmic Transport to Spindle Assembly and Beyond

**DOI:** 10.3389/fcell.2015.00082

**Published:** 2016-01-11

**Authors:** Tommaso Cavazza, Isabelle Vernos

**Affiliations:** ^1^Cell and Developmental Biology, Centre for Genomic Regulation, The Barcelona Institute of Science and TechnologyBarcelona, Spain; ^2^Universitat Pompeu FabraBarcelona, Spain; ^3^Institució Catalana de Recerca I Estudis AvançatsBarcelona, Spain

**Keywords:** spindle, RanGTP, microtubule, cell division, importin, SAF, nucleo-cytoplasmic transport, exportin

## Abstract

The small GTPase Ran regulates the interaction of transport receptors with a number of cellular cargo proteins. The high affinity binding of the GTP-bound form of Ran to import receptors promotes cargo release, whereas its binding to export receptors stabilizes their interaction with the cargo. This basic mechanism linked to the asymmetric distribution of the two nucleotide-bound forms of Ran between the nucleus and the cytoplasm generates a switch like mechanism controlling nucleo-cytoplasmic transport. Since 1999, we have known that after nuclear envelope breakdown (NEBD) Ran and the above transport receptors also provide a local control over the activity of factors driving spindle assembly and regulating other aspects of cell division. The identification and functional characterization of RanGTP mitotic targets is providing novel insights into mechanisms essential for cell division. Here we review our current knowledge on the RanGTP system and its regulation and we focus on the recent advances made through the characterization of its mitotic targets. We then briefly review the novel functions of the pathway that were recently described. Altogether, the RanGTP system has moonlighting functions exerting a spatial control over protein interactions that drive specific functions depending on the cellular context.

## Historical perspective on the chromatin dependent MT assembly pathway

The first hints of the existence of a chromosome-dependent MT assembly mechanism in the dividing cell were obtained in the 1970–1980s when several groups reported that MT nucleation occurred close to or at the kinetochores (McGill and Brinkley, 1975; Telzer et al., 1975; Witt et al., 1980; De Brabander et al., 1981) and a spindle like structure formed around lambda DNA injected into metaphase arrested *Xenopus* eggs (Karsenti et al., 1984). In 1996, DNA coated beads were shown to trigger bipolar spindle formation when incubated in *Xenopus* egg extracts (Heald et al., 1996), providing further support to the idea that chromatin carries all the information required to direct MT assembly and organization in the M-phase cytoplasm. Shortly after, the identification of the small Ran GTPase as driver of chromatin-dependent MT assembly in the M-phase cytoplasm provided a major breakthrough to understand the underlying mechanism (Carazo-Salas et al., 1999; Kalab et al., 1999; Ohba et al., 1999; Wilde and Zheng, 1999; Zhang et al., 1999). Today, we know that the chromosomes drive MT assembly and organization into a bipolar spindle in a RanGTP dependent manner in most cells (Karsenti and Vernos, 2001; Rieder, 2005).

In this mini-review we will describe briefly how the RanGTP system regulates the nucleo-cytoplasmic shuttling of components in interphase and, after NEBD, the activity and/or localization of specific factors to drive spindle assembly. We will briefly review our current knowledge on the identity and function of RanGTP regulated factors and the recent advances on understanding novel mechanisms regulated by RanGTP. Finally we will provide an overview of the regulation of the RanGTP pathway itself during mitosis, its conservation in different organisms and cell types, and its role in other cellular functions. For additional information we refer the reader to excellent reviews (Ciciarello et al., 2007; O'Connell and Khodjakov, 2007; Clarke and Zhang, 2008; Kalab and Heald, 2008; Roscioli et al., 2010; Forbes et al., 2015).

## The nucleo-cytoplasmic transport and the small GTPase ran

**Figure 1:**
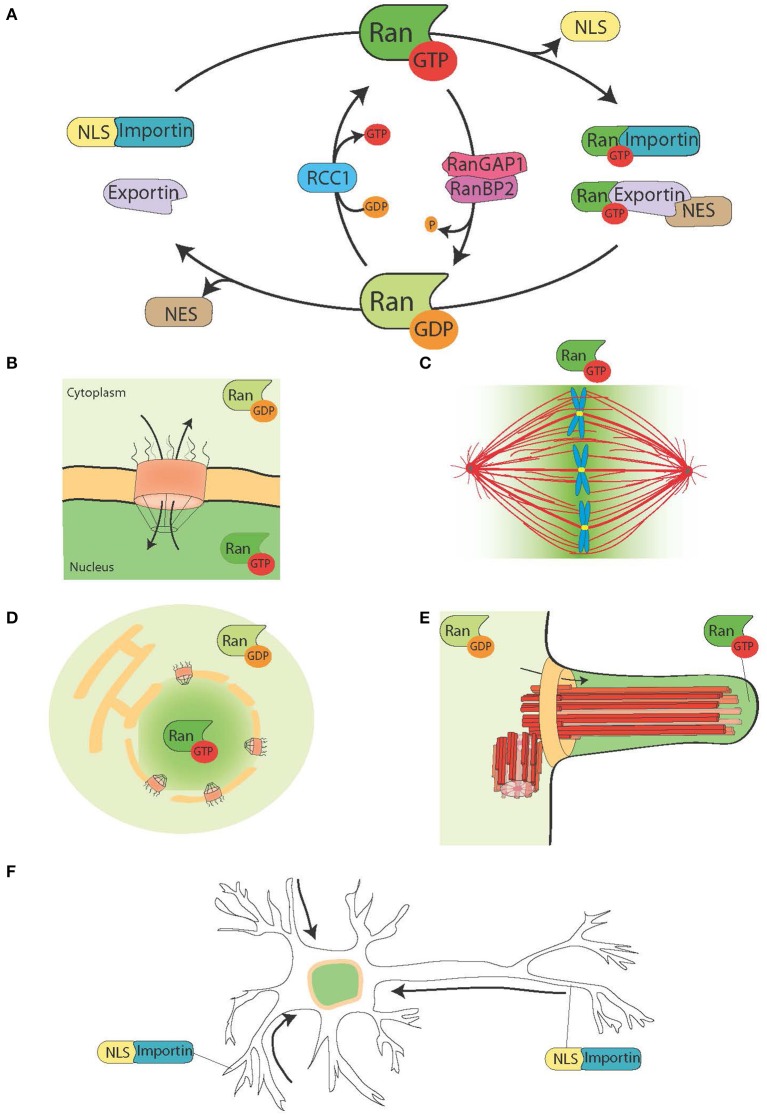
**The Ran system and its moonlighting functions**. **(A)** Schematic representation of the Ran system for the spatial control of NLS and NES carrying proteins. In cells Ran is found in two forms, RanGTP (green), and RanGDP (light green). RCC1 (light blue) promotes the exchange of GDP to GTP, while RanGAP1-RanBP2 (in pink and purple) promote the hydrolysis of GTP into GDP. RanGTP binds to the importins (turquoise green) and exportins (light purple). Exportins in complex with RanGTP can associate to the NES-proteins (in brown). On the other hand, the binding of RanGTP to importins trigger their dissociation from NLS-proteins (yellow). **(B)** During interphase, the Ran system controls the nucleo-cytoplasmic shuttling of proteins, because RanGTP is predominant in the nucleoplasm and RanGDP is predominant in the cytoplasm (Sorokin et al., 2007). **(C)** During mitosis the association of RCC1, the RanGEF, with the chromosomes defines a gradient of RanGTP concentrations that promote the release of SAFs and MT nucleation around the chromatin. The Ran system is converted into a pathway for MT assembly and organization that is essential for mitotic spindle assembly. The RanGTP pathway depends on the establishment of a concentration gradient of RanGTP that peaks around the chromosomes (Kalab et al., 2002; Caudron et al., 2005). **(D)** At the end of mitosis, the Ran system also regulates nuclear membrane and NPC reassembly by controlling membrane fusion and releasing NPC components (Walther et al., 2003; Harel et al., 2003). **(E)** In ciliated cells RanGTP accumulates in the cilioplasm and promotes the transport and accumulation of Kif17 and retinis pigmentosa 2 to the cilioplasm (Dishinger et al., 2010; Fan et al., 2011; Hurd et al., 2011). **(F)** In neurons many SAFs have a function. Furthermore, importins localize to the dendritic synaptic space and are involved in the transport of cargos to the nucleus (Jordan and Kreutz, 2009; Panayotis et al., 2015). The Ran system is also active in the axon of the sciatic nerve, where upon injury importins promote the transport of cargos toward the neuron cell body (Hanz et al., 2003; Yudin et al., 2008).

Eukaryotic cells are compartmentalized and have specific transport systems for the communication between the cytoplasm and the different membrane-bound organelles. The nucleo-cytoplasmic transport system is essential to connect functionally the transcription of the genome that occurs within the nucleus, with protein translation that takes place in the cytoplasm (Figures 1A,B). The transport of molecules in and out of the nucleus occurs through the nuclear pore complex (NPC), a big protein complex of ~60 MDa inserted into the nuclear membrane (Sorokin et al., 2007). Small cargos (< 40 kDa) diffuse rapidly through the NPC. Instead, proteins larger than 40 kDa require an active transport through the NPC that involves soluble nuclear transport receptors (NTRs) that belong to the karyopherin-β protein family. NTRs that facilitate the transport of cargo proteins into the nucleus are called importins and interact with their cargo through a nuclear localization signal (NLS) rich in basic residues. NTRs facilitating the export of proteins out of the nucleus are called exportins and interact with their cargo through a nuclear export signal (NES) rich in hydrophobic residues such as leucine. The karyopherin-β importin β1 often interacts with the cargo through an adaptor of the importin α family (Sorokin et al., 2007). Importin α binds directly to the NLS of the cargo protein and to importin β1 through an IBB domain (importin β binding domain), leading to the formation of a trimeric complex.

NTRs associate with the small GTPase Ran that acts as a molecular switch. In its GTP bound form, Ran (RanGTP) interacts with karyopherin-β proteins with high affinity, while it dissociates in its GDP bound form (RanGDP). RanGTP binding to importins and exportins have very different consequences: it stabilizes the exportin-cargo interaction whereas it destabilizes the importin-cargo interaction (Figure 1A).

The RanGEF (guanine nucleotide exchange factor) RCC1 associates with the chromatin inside the nucleus, whereas RanGAP (GTPase activating protein) is cytoplasmic. As a consequence the predominant form of Ran in the nucleus is bound to GTP, while in the cytoplasm it is bound to GDP. Thereby NLS proteins transported to the nucleus by importins are released and accumulate in the nucleoplasm, whereas NES proteins in complex with exportin-RanGTP are transported out of the nucleus (Figures 1A,B).

Although the nucleo-cytoplasmic transport is no longer needed when a cell enters into mitosis, its complex molecular machinery is recycled to promote MT assembly around the chromatin and to direct the organization of the bipolar spindle (Clarke and Zhang, 2008).

## The RanGTP pathway during cell division

As RCC1 remains associated with the chromatin after NEBD, RanGTP is highly enriched in the proximity of the chromosomes. As RanGTP diffuses away from the chromatin, RanGAP in the cytoplasm converts it into RanGDP (Figure 1C). The resulting gradient has been directly visualized in cells and *Xenopus* egg extracts (Kalab et al., 2002, 2006) and its properties in MT nucleation and stabilization tested and modeled (Caudron et al., 2005). Like in interphase, this system provides a spatial control over the stability of NTRs-cargo complexes. The cargos are NLS and/or NES containing proteins with specific functions related to spindle assembly and function. The NLS-proteins with a role in spindle assembly have been named SAFs (Spindle Assembly Factors).

The discovery and characterization of the RanGTP pathway prompted a re-examination of the Search and Capture model for spindle assembly proposed in 1986 (Kirschner and Mitchison, 1986). This model postulates that centrosomal MTs grow and shrink exploring the cytoplasmic space until a stochastic encounter with a kinetochore promotes their capture and attachment. However, it has been now clearly established that animal cells experimentally deprived from their centrosomes do assemble a functional mitotic spindle (Debec et al., 1995; Khodjakov et al., 2000). Moreover, mathematical simulations suggested that the Search and Capture mechanism could not account for the short division time observed in most animal cells (Wollman et al., 2005). By promoting MT nucleation and stabilization in the proximity of the chromosomes, the RanGTP pathway most certainly favors MT capture by the kinetochores increasing the efficiency of the Search and Capture mechanism. However, the role of the RanGTP pathway must go beyond MT capture by the kinetochores and kinetochore-fiber (K-fiber) formation since it also promotes MT organization in the absence of chromosomes, kinetochores, and K-fibers (Carazo-Salas et al., 1999). The identification of the direct and indirect RanGTP targets in the M-phase cytoplasm is therefore an essential step to fully understand the several roles this pathway fulfills during cell division.

## Understanding the RanGTP pathway through the identification and functional characterization of its targets

A direct read out of the role of RanGTP in the M-phase cytoplasm was obtained in *Xenopus* egg extracts devoid of chromatin and centrosomes. Addition of RanGTP to these extracts is indeed sufficient to trigger MT nucleation, promote MT stabilization, and induce the organization of MT assemblies named mini-spindles (Carazo-Salas et al., 1999, 2001). Therefore, one or more SAFs maybe involved in these different events.

Since the identification of the first SAFs in 2001 (Gruss et al., 2001; Nachury et al., 2001; Wiese et al., 2001; Clarke and Zhang, 2008; Meunier and Vernos, 2012), the number of proteins controlled by RanGTP in mitosis has been slowly growing and several novel SAFs were identified recently (CDK11, CHD4, ISWI, Kif14, Kif2a, MCRS1, Mel28, Anillin, APC; Silverman-Gavrila et al., 2008; Yokoyama et al., 2008, 2009, 2014; Dikovskaya et al., 2010; Meunier and Vernos, 2011; Samwer et al., 2013; Wilbur and Heald, 2013). Currently, 22 proteins have been validated as SAFs (Table 1). In addition, a number of proteins with established roles in various aspects of spindle assembly are nuclear and could therefore be targets for RanGTP regulation (i.e., Kif4a/Klp1, Ino80, Reptin), but further studies should address this possibility.

**Table 1:** **Spindle assembly factors**.

	**Protein name**	**Mitotic function**	**Mitotic localization**	**Interphase function**	**Interphase localization**	**Importin**	**References**
**Chromatin Remodeling**	CHD4	Stabilizes MTs	MTs and DNA	Chromatin Remodeling complex (NuRD), to inhibit transcription; also in DNA damage response	Nucleus	α1–β1	Oshaughnessy and Hendrich, 2013; Stanley et al., 2013; Yokoyama et al., 2013
	ISWI1	Stabilizes MTs, mostly in anaphase	Centrosomes, Spindle poles and DNA	ATPase subunit of Chromatin remodeling complex; involved in DNA repair, DNA Replication, Chromatin structure	Nucleus	α1–β1	Yokoyama et al., 2009; Toto et al., 2014
	MCRS1	Protects MT -end, favors Chromatin MT assembly and K-fiber formation	Spindle poles, K-fibers—ends	rRNA production; Ino80 complex, NSL complex	Nucleolar	β1	Shimono et al., 2005; Raja et al., 2010; Watanabe and Peterson, 2010; Meunier and Vernos, 2011
**Kinesins**	Kif14-NabKin	+end directed motor, important for chromosome congression and cytokinesis	MTs	Focal adhesion (Rap1a-Radil signaling)	Cytoplasm, MTs and Centrosome	β1	Zhu et al., 2005; Carleton et al., 2006; Ahmed et al., 2012; Samwer et al., 2013
	Kid (Kif22)	+end directed chromokinesin, important for for chromosome arm congression	MTs and Chromatin	n.d.	Nucleus	α1-β1	Tokai et al., 1996; Tahara et al., 2008
	HSET/XCTK2/KIFC1	-end directed kinesin, important for pole focusing	MTs	Endocytic transport and DNA transport	Nucleus	α1-β1	Walczak et al., 1997; Ems-McClung et al., 2004; Nath et al., 2007; Farina et al., 2013
	Kif2a	MT depolymerizing kinesin. Important for spindle length, pole coalescence, and chromosome congression	MTs	? Primary cilia disassembly; axonal pruning	Centrosome	α1-β1	Maor-Nof et al., 2013; Wilbur and Heald, 2013; Eagleson et al., 2015; Miyamoto et al., 2015
**Nuclear Pore Complex**	Mel28/ELYS	Ran Dependent MT nucleation, interacts with γTubulin	Spindle poles, kinetochores	NPC re-assembly	NPC	β1; transp.	Rasala et al., 2006; Lau et al., 2009; Yokoyama et al., 2014
	Nup107-160 complex	Ran Dependent MT nucleation, interacts with γTubulin, CPC localization	Spindle poles, kinetochores	NPC	NPC	β1; transp.	Orjalo et al., 2006; Lau et al., 2009; Platani et al., 2009; Mishra et al., 2010
	Nup98	Inhibits MCAK activity	n.d.	NPC	NPC	β1; transp.	Lau et al., 2009; Cross and Powers, 2011
	Rae1	Spindle organization; counteracts NuMa function	Spindle poles	Nucleoporine, involved in RNA export, interacts with Nup98	NPC	β1	Pritchard et al., 1999; Blower et al., 2005; Wong et al., 2006
	Lamin B3^*^	Spindle organization, supposedly through the spindle matrix	MTs	Mechanical properties of the nucleus, but also DNA replication, DNA transcrption and DNA damage	Nucleus and NE	α1-β1	Tsai et al., 2006; Adam et al., 2008; Osmanagic-Myers et al., 2015
**Others**	TPX2	MT nucleation, MT bundling, AurA activation	MTs	Binds DNA; post mitotic neurons MT assembly	Nucleus	α1-β1	Wittmann et al., 2000; Gruss et al., 2001; Mori et al., 2009; Neumayer et al., 2014; Scrofani et al., 2015
	NuMA	Spindle pole formation and Spindle positioning	MTs	Nuclear matrix; Chromatin organization; Splicing; Recombination upon DNA damage	Nucleus	β1	Compton and Cleveland, 1993; Zeng et al., 1994; Gaglio et al., 1995; Nachury et al., 2001; Wiese, 2001; Abad et al., 2007; Radulescu and Cleveland, 2010; Kiyomitsu and Cheeseman, 2012; Vidi et al., 2014
	NuSAP	Important for MT stabilization and crosslinking, favors MT assembly in proximity of chromatin	MTs and chromatin	n.d.	Nucleolar	α1-β1; -β7	Raemaekers, 2003; Ribbeck et al., 2006, 2007
	HURP	Stabilizes and bundles MTs, specially k- fibers	k- fibers	Adherent Juntions in Epithelial cells	Mostly Cytoplasm, but it shuttles	β1	Tsou et al., 2003; Laprise et al., 2004; Koffa et al., 2006; Sillje et al., 2006
	TACC3	MT elongation and K-fiber formation	Spindle poles and MTs	mRNA translation; Sequesters transcription factor FOG1; Hypoxia Inducible Factor complex; +Tips MTs	Cytoplasmic, MTs	β1, not clear data	Stebbins-Boaz et al., 1999; Gergely et al., 2000; Garriga-Canut and Orkin, 2004; Peset et al., 2005; Albee et al., 2006; Guo et al., 2013; Nwagbara et al., 2014
	CDK11	Centrosome maturation and MT stability)	Spindle poles/centrosomes	Many; i.e., mRNA splicing	Nucleus and Centrosomes	β1	Petretti et al., 2006; Yokoyama et al., 2008; Malumbres, 2014
	Xnf7^**^	Stabilizes and bundles MTs; inhibits APC/C at anaphase on set	MTs	Transcription, E3 Ub ligase	Nucleus	β1	Etkin et al., 1997; Casaletto, 2005; Maresca et al., 2005; Beenders et al., 2007; Sinnott et al., 2014
	APC	Bundles MTs	MTs and kinetochores	Many: Transcription, cell migration, Wnt signaling pathway, inhibits DNA replication	Cytoplasmic, MTs	β1	Dikovskaya et al., 2001, 2004, 2010, 2012; Perchiniak and Groden, 2011
	Crb3-Clp1^***^	Not charcterized function, disorganized spindles	Spindle poles	n.d.	Cilia and Nuclear membrane	β1	Fan et al., 2007
	Anillin	Cytokinesis, membranes elongation in anaphase	Cell cortex	Sequestered to the nucleus, if in the cytoplasm is deleterious	Nucleus	α1-β1	Field and Alberts, 1995; Silverman-Gavrila et al., 2008;Kiyomitsu and Cheeseman, 2013

* Only amphibians have Lamin B3;

** XL name (By Blast TRIM69, 43% identity, Trim69i impairs spindle assembly);

*** Crb3, no Clp1.

Interestingly, the functional characterization of some of the SAFs is providing mechanistic insights into the RanGTP pathway functions in the dividing cell. The mechanism by which RanGTP promotes MT nucleation *de novo* in the M-phase cytoplasm was recently described (Scrofani et al., 2015). By releasing TPX2 from importins, RanGTP promotes its interaction with Aurora A and with a RHAMM-NEDD1-γTURC (γTubulin Ring Complex) complex. In this new complex the activated Aurora A phosphorylates NEDD1, an essential requirement for MT nucleation. Another SAF, Mel28, was shown to interact with the γTuRC and it was proposed to play a role in RanGTP dependent MT nucleation (Yokoyama et al., 2014). The potential cooperation of Mel28 with the TPX2-dependent pathway described above remains to be established.

The RanGTP pathway also contributes to centrosome maturation and its MT assembly activity (Carazo-Salas et al., 2001). In fact two SAFs, CDK11, and Mel28 were shown to favor MT assembly at the centrosome (Yokoyama et al., 2008, 2014).

The identification and characterization of another SAF, MCRS1, has revealed a novel and important mechanism for the regulation of K-fiber MT minus-end dynamics (Meunier and Vernos, 2011) and novel insights on the roles of the RanGTP pathway in spindle assembly and cell division (Meunier and Vernos, 2012). MCRS1, in complex with members of the chromatin modifier KAT8-associated nonspecific lethal (KANSL) complex (Meunier et al., 2015), is targeted to the minus-end of RanGTP-dependent MTs protecting them from depolymerisation. Within the spindle MCRS1 also associates specifically with the minus-ends of K-fiber MTs and regulates their depolymerisation rate playing an essential role in K-fiber dynamics and chromosome alignment (Meunier and Vernos, 2011; Meunier et al., 2015). The specific association of MCRS1 with the MTs nucleated by the RanGTP dependent pathway also suggests that these MTs have specific characteristics that distinguish them from the MTs nucleated by the centrosomes. If this turns out to be true, the chromosomal MTs would not be merely a local supply of MTs favoring an efficient Search and Capture mechanism, but they could provide essential unique functionalities required for the assembly and function of the bipolar spindle (Meunier et al., 2015).

Recently the MT depolymerizing kinesin Kif2a was shown to be regulated by RanGTP in mitosis, revealing an important mechanism for the scaling of the spindle to the cell size during the early development of *Xenopus* embryos (Wilbur and Heald, 2013). Kif2a is maintained inactive by importin α until stage 8 of embryonic development. As the soluble concentration of importin α decreases, Kif2a is released and function as a MT depolymerase promoting spindle shortening.

Although, most of the SAFs identified so far were found to play a role in the early phases of cell division, a number of recent reports indicate that the RanGTP pathway has other essential roles not directly related to spindle assembly. Indeed, the characterization of the SAF ISWI suggests functions for the RanGTP pathway during anaphase (Yokoyama et al., 2009).

Multiple lines of research also indicate that it plays a role in spindle positioning. Indeed, before entry into anaphase, the RanGTP gradient restricts the localization of the LGN-NuMa complex to cell cortex areas further away from the chromosomes, contributing to the control of spindle position and orientation (Kiyomitsu and Cheeseman, 2012).

In addition, RanGTP also regulates non-MT related targets. Indeed, it controls Anillin localization and triggers asymmetric membrane elongation during anaphase, defining spindle positioning at the center of the dividing cell (Silverman-Gavrila et al., 2008; Kiyomitsu and Cheeseman, 2012). Finally, during cytokinesis the RanGTP pathway regulates the activity of the kinesin Kif14/Nabkin in actin bundling (Carleton et al., 2006; Samwer et al., 2013) and coordinates nuclear membrane and NPC reassembly (Harel et al., 2003; Walther et al., 2003; Ciciarello et al., 2010; Roscioli et al., 2010; Forbes et al., 2015; Figure 1D).

It is therefore clear that the identification and functional characterization of the RanGTP mitotic targets is providing novel insights into the mechanism of spindle assembly and cell division. However, it is unclear whether many or only a few more RanGTP targets remain to be identified. This number could be potentially high as the number of nuclear proteins is in the order of hundreds or thousands (Dellaire et al., 2003), at least one order of magnitude above the current number of known RanGTP targets in the dividing cell (Table 1).

Most of the proteomic studies aimed at identifying novel SAFs have focused on importins α1 and β1 (Nachury et al., 2001; Wiese et al., 2001; Yokoyama et al., 2008), which are two of the most abundant importins in Xenopus egg extracts (Bernis et al., 2014; Wuhr et al., 2014). However, there are five additional α-importins and eight additional β-importins in humans (Cautain et al., 2015).

Although still scarce, some data indicate that indeed other importins also play a role during cell division. The RanGTP regulation of NuSAP was shown to depend on importin-β1 and importin-7 (Ribbeck et al., 2006) and that of Mel28, Nup107-160, and Nup98 on importin-β1 and transportin/importin-β2 (Lau et al., 2009). Transportin was also specifically shown to negatively regulate spindle assembly and nuclear membrane and NPC reassembly (Bernis et al., 2014). However, there are no described mitotic factors exclusively regulated by importin-7 or transportin.

The characterization of possible transportin specific targets and, more generally, of the other importins α and β represents an open field for exploration. This could be important to understand the regulation of the RanGTP pathway, especially considering that importins expression patterns change significantly in different developmental stages and tissues (Hosokawa et al., 2008).

## Regulation of the RanGTP system during cell division

Beyond the specificities of NTR-SAF interactions, several mechanisms may directly impinge upon the RanGTP pathway during cell division. Several data suggest that RCC1 itself is a key component under fine regulation. Human cells have three isoforms of RCC1, that are expressed in a tissue specific manner (Hood and Clarke, 2007). The isoforms differ at their N-terminus, a region involved in importin binding and regulated by phosphorylation, which was proposed to influence chromosome-coupled RanGTP production (Hood and Clarke, 2007; Li et al., 2007). Moreover, the level of RCC1 expression also varies in different cells and correlates with the steepness of the RanGTP gradient (Hasegawa et al., 2013). This may have important consequences as it was proposed that the steepness of the RanGTP gradient determines the length of prometaphase and metaphase which in turn may be relevant for chromosome segregation fidelity (Silkworth et al., 2012; Hasegawa et al., 2013).

Other mechanisms, such as post-translational modifications and alternative splicing are also potential strategies to control the NLS of SAFs. However, these mechanisms would rather affect a particular protein than the whole RanGTP pathway.

Recently, an alternative mechanism for the regulation of SAFs independently of RanGTP was proposed. The targeting of the Golgi protein GM130 to fragmented Golgi membranes in mitosis may compete out locally TPX2 from the importin α1 binding, thus favoring MT assembly in the vicinity of Golgi fragments (Wei et al., 2015). This competition-based mechanism could be another strategy to locally control SAFs sequestered by importins.

## The role of other components of the nucleo-cytoplasmic shuttling machinery during mitosis

The binding of RanGTP to exportins stabilizes its interaction with NES-cargo proteins. The major exportin, CRM1, was shown to be involved in the targeting of NES-proteins to the kinetochore or the centrosomes. At the kinetochore, CRM1 recruits the RanBP2-RanGAP1-SUMO complex that is required for the interaction between MTs and the kinetochore (Arnaoutov et al., 2005). However, it is still mechanistically unclear how this complex favors the MT-kinetochore interaction (Forbes et al., 2015). CRM1 also promotes the recruitment of RanGAP1-RanBP2 to the spindle in a RanGTP dependent manner (Wu et al., 2013) and it is involved in tethering the Chromosome Passenger Complex to the centromere through its direct interaction with survivin (Knauer et al., 2006). CRM1 has also been shown to promote the recruitment of BRCA1 and pericentrin to the mitotic centrosomes, thus promoting the MT assembly activity of the centrosomes (Liu et al., 2009; Brodie and Henderson, 2012). Recently, the transcriptional repressor Bach1 was found to play a role in chromosome arm alignment during mitosis and to be excluded from the chromosomes during metaphase in a CRM1-dependent way (Li et al., 2012).

However, the significance of these targeting events is not entirely clear mechanistically (Yokoyama and Gruss, 2013). A major problem is that during mitosis the putative role of exportin mediated interactions may be difficult to untangle from that of importin mediated interactions, as they involve proteins having both NES and NLS [i.e., Pericentrin (Liu et al., 2010)]. Nevertheless, it seems evident that the RanGTP regulation of CRM1 has several roles during mitosis and it will be interesting to test whether other exportins are also important for mitotic events.

## Conservation of the RanGTP pathway in dividing cells

In the last 15 years the RanGTP pathway has been studied in several organisms and cell types. It was found to present variations on some details or in some cases to be unnecessary. Indeed, in some meiotic systems the contribution of the RanGTP pathway appears to be non-essential. For instance, *Drosophila* spermatocytes can assemble the meiosis I spindle in the complete absence of chromosomes (Bucciarelli et al., 2003). The assembly of the acentrosomal spindle of meiosis I in mice and frogs oocytes was also shown to be only partially dependent on the RanGTP pathway, although the pathway is strictly essential for spindle assembly during meiosis II (Dumont et al., 2007).

Even in systems relying on RanGTP for spindle assembly there are some variations at least at the level of the machinery. For instance, TPX2, which is essential in frogs and mammals, is not present in *Caenorhabditis elegans* and *Drosophila melanogaster*. Although proteins with some of the TPX2 characteristics have been identified in these systems (Ozlu et al., 2005; Goshima, 2011), they lack essential features of TPX2, like an NLS that is at the basis of the RanGTP regulation. This example indicates that the effectors of the RanGTP pathway might vary from system to system, although the main principles are probably maintained and conserved.

## The RanGTP pathway: a moonlighting pathway with a role in several cellular functions

The RanGTP pathway is an example of a whole pathway that accomplishes essential functions in different parts of the cell cycle. In interphase, it orchestrates the nucleo-cytoplasmic transport, while in mitosis it drives spindle assembly and later nuclear membrane and NPC reassembly (Figures 1B–D). Individual proteins that have different functions at different times are defined as moonlighting proteins (Jeffery, 1999). The RanGTP pathway could therefore be an example of a moonlighting pathway.

The RanGTP pathway is particularly interesting, because it shows how the function of a protein depends on its context: most of the SAFs have nuclear functions and are kept separated from tubulins and others cytoskeleton proteins during interphase. Upon NEBD, the general context changes and the SAFs exert important functions related to the MTs.

Some data point toward a moonlighting function of the RanGTP pathway in cilia formation and in transport into the cilium. RanGTP has been shown to control the accumulation of Kif17 and retinis pigmentosa 2 to the cilioplasm (Dishinger et al., 2010; Hurd et al., 2011), where RanGTP is concentrated (Fan et al., 2011). The current working model is that the RanGTP pathway orchestrates the transport of cargos carrying a cilia localization signal through the cilia pore complex, which has been proposed to be located at the base of the cilium (Kee et al., 2012; Figure 1E). However, further studies are needed to understand how the RanGTP gradient is established in cilia and what other cargos it transports into the cilia.

Interestingly, the RanGTP pathway moonlights also in differentiated neurons, where many SAFs also have a function [TPX2, MCRS1, NuMa, Rae1, HSET (Ferhat et al., 1998; Davidovic et al., 2006; Mori et al., 2009; Tian et al., 2011; Pannu et al., 2015)]. Furthermore importins α and β accumulate at the dendritic synaptic space and have a role in the transport of cargos from the synapses to the nucleus (Jordan and Kreutz, 2009; Panayotis et al., 2015). Finally, a RanGTP regulated mechanism has been shown to be at play in response to sciatic nerve injuries (Hanz et al., 2003; Yudin et al., 2008; Figure 1F).

## Conclusions

The identification of the role played by the RanGTP pathway during cell division occurred more than 15 years ago. We know now that the RanGTP pathway has additional functions and could be considered a moonlighting pathway controlling various important cellular processes (Figure 1). During cell division it drives essential mechanisms that we start to understand thanks to the identification and functional characterization of its direct targets. However, several open questions still need to be addressed. The total number of SAFs is difficult to anticipate and therefore we do not know how many still remain to be identified. Furthermore, most of our current knowledge is restricted to the role of only some components of the nucleo-cytoplasmic transport machinery. For instance, very little is currently known about the putative role in cell division of the different importins present in the human cell. Specific importins may regulate the activity of novel SAFs and their different expression patterns in different cell types and tissues may provide a relevant combinatorial mechanism. We also know little on the putative role of the components of the export machinery in spindle assembly and in the other novel functions of the pathway. Although, there are data suggesting various points of regulation of the pathway itself, the consequences on cell division and other processes are not clear yet, nor how it may be adapted to the requirements of different cell types or tissues. The study of the RanGTP pathway will certainly provide exciting new insights in the next few years, revealing some essential mechanisms for cell organization and function.

## Author contributions

IV and TC wrote the manuscript, TC prepared the table and figure.

## Funding

TC was supported by the Spanish Ministry of Economy and Competitiveness (MINECO) through the FPI fellowship BES-2010-031355. Work in the Vernos lab was supported by the Spanish ministry grants BFU2009-10202 and BFU2012-37163, co-financed by the European Regional Development Fund (ERDF/FEDER). We also acknowledge support of the Spanish Ministry of Economy and Competitiveness, “Centro de Excelencia Severo Ochoa 2013-2017,” SEV-2012-0208.

### Conflict of interest statement

The authors declare that the research was conducted in the absence of any commercial or financial relationships that could be construed as a potential conflict of interest.

## Abbreviations

γTuRC: γTubulin Ring Complex
K-Fiber: Kinetochore-Fiber
KANLS: KAT8-associated nonspecific lethal complex
MT: Microtubule
NEBD: Nuclear Envelope Breakdown
NES: Nuclear Export Signal
NLS: Nuclear Localization Signal
NPC: Nuclear Pore Complex
NTR: Nuclear Transport Receptor
RanGAP: Ran GTPase Activating Protein
RanGEF: Ran Guanine nucleotide Exchange Factor
SAF: Spindle Assembly Factor.
